# Short-Chain Fatty Acids Reduced Renal Calcium Oxalate Stones by Regulating the Expression of Intestinal Oxalate Transporter SLC26A6

**DOI:** 10.1128/mSystems.01045-21

**Published:** 2021-11-16

**Authors:** Yu Liu, Xi Jin, Yucheng Ma, Zhongyu Jian, Zhitao Wei, Liyuan Xiang, Qun Sun, Shiqian Qi, Kunjie Wang, Hong Li

**Affiliations:** a Department of Urology, Institute of Urology (Laboratory of Reconstructive Urology), West China Hospital, Sichuan University, Chengdu, Sichuan, People’s Republic of China; b Key Laboratory of Bio-resources and Eco-environment of the Ministry of Education, College of Life Sciences, Sichuan University, Chengdu, Sichuan, People’s Republic of China; c State Key Laboratory of Biotherapy and Cancer Center, West China Hospital, Sichuan University, Chengdu, Sichuan, People’s Republic of China; Vanderbilt University Medical Center

**Keywords:** renal calcium oxalate stones, short-chain fatty acids, intestinal oxalate transporters, gut microbiota, oxalate

## Abstract

Renal calcium oxalate (CaOx) stone is a common urologic disease with a high prevalence and recurrence rate. However, short-chain fatty acids (SCFAs) are less often reported in the prevention of urolithiasis. This study aimed to explore the effect of SCFAs on the renal CaOx stone formation and the underlying mechanisms. Ethylene glycol was used to induce renal CaOx crystals in rats. SCFAs (acetate, propionate, or butyrate) were added as supplements to the drinking water with or without antibiotics. Because intestinal oxalate transporters SLC26A6 and SLC26A3 regulate the excretion and absorption of oxalate in the intestine, we injected adeno-associated virus 9 (AAV9)-SLC26A6-shRNA (short hairpin RNA) and AAV9-SLC26A3 into the tail vein of rats to suppress SLC26A6 and overexpress SLC26A3 expression in the intestine, respectively, to explore the role of SLC26A3 and SLC26A6 (SLC26A3/6) in the reduction of renal CaOx crystals induced by SCFAs. Results showed that SCFAs reduced renal CaOx crystals and urinary oxalate levels but, however, increased the abundance of SCFA-producing bacteria and cecum SCFA levels. SCFA supplements still reduced renal crystals and urinary oxalate after gut microbiota depletion. Propionate and butyrate downregulated intestinal oxalate transporter SLC26A3 expression, while acetate and propionate upregulated SLC26A6 expression, both *in vivo* and *in vitro*. AAV9-SLC26A3 exerted a protective effect against renal crystals, while AAV9-SLC26A6-shRNA contributed to the renal crystal formation even though the SCFAs were supplemented. In conclusion, SCFAs could reduce urinary oxalate and renal CaOx stones through the oxalate transporter SLC26A6 in the intestine. SCFAs may be new supplements for preventing the formation of renal CaOx stones.

**IMPORTANCE** Some studies found that the relative abundances of short-chain-fatty-acid (SCFA)-producing bacteria were lower in the gut microbiota of renal stone patients than healthy controls. Our previous study demonstrated that SCFAs could reduce the formation of renal calcium oxalate (CaOx) stones, but the mechanism is still unknown. In this study, we found that SCFAs (acetate, propionate, and butyrate) reduced the formation of renal calcium oxalate (CaOx) crystals and the level of urinary oxalate. Depleting gut microbiota increased the amount of renal crystals in model rats, and SCFA supplements reduced renal crystals and urinary oxalate after gut microbiota depletion. Intestinal oxalate transporter SLC26A6 was a direct target of SCFAs. Our findings suggested that SCFAs could reduce urinary oxalate and renal CaOx stones through the oxalate transporter SLC26A6 in the intestine. SCFAs may be new supplements for preventing the formation of renal CaOx stones.

## INTRODUCTION

Renal stone is one of the most common urologic diseases (1), and the prevalence ranges from 5% to 19% worldwide (2, 3). After the first affliction, renal stones of one-third of patients will be recurrent within 5 years, and patients with recurrent kidney stones suffer an elevated risk of further episodes (4, 5). Calcium oxalate (CaOx) accounts for a 60% to 90% component of kidney stones (6–8). Studies showed that the level of oxalate in the urine was higher in renal CaOx stone patients than in healthy controls, which suggests that high urinary oxalate is a potential risk factor for stones (9–11).

Urinary oxalate is mainly derived from various precursors of the endogenous metabolism in the liver and exogenous oxalate from diet. Studies reported that the gut took part in the oxalate metabolism by absorbing and excreting oxalate via oxalate transporters (12–15). The oxalate transporter SLC26A6, mainly expressed in the small intestine, is a major oxalate-excreting transporter. In contrast, SLC26A3 can mediate oxalate absorption in ileum, cecum, and colon (16–18). Studies have demonstrated that mice with a defective SLC26A6 transporter developed hyperoxaluria and CaOx stones (19, 20). SLC26A3 gene knockout mice showed a lower blood and urine oxalate level (21). Thus, SLC26A3 and SLC26A6 (SLC26A3/6) are promising targets to decrease the level of urinary oxalate. However, the mechanism by which cells regulate the expression level of oxalate transporters in the intestine has not been completely clarified.

Oppositely, oxalate can be removed from the intestine by the gut microbiota, e.g., Oxalobacter formigenes. Recently, several studies showed that gut microbiota is essential in renal stone formation (22). Interestingly, the urinary oxalate level is lower in people colonized with *O. formigenes* than in those not colonized (23, 24). However, probiotics containing *O. formigenes* cannot decrease urinary oxalate levels significantly (25, 26). We previously found that the relative abundance of bacteria producing short-chain fatty acids (SCFAs) was lower in the gut microbiota of renal stone patients than healthy people (27). Additionally, SCFAs could reduce the formation of renal CaOx stones, but the mechanism is still unknown (27). SCFAs are products from bacterial fermentation of dietary fiber and cannot be generated by the metabolism in humans (28). Multiple studies highlighted the benefits of SCFAs of mediating host metabolism and immunity (29–32). Dysbiosis of SCFA metabolism is associated with various diseases, such as diabetes mellitus, colorectal cancer, and kidney disease (33–35). In the human gut, the most abundant SCFAs from the fermentation of microbes are acetic acid, propionic acid, and butyric acid, which can function as signal molecules in various pathways (28). SCFAs could inhibit histone deacetylases or activate GPR43 to perform anti-inflammatory or immune-suppressive functions (28). For example, the butyrate-induced upregulation of glucagon-like peptide 1 and peptide YY may be important in preventing or treating obesity and insulin resistance (36). A recent study showed that vinegar, the primary component of which is acetic acid (5% to 8%), can prevent kidney stone recurrence by enhancing urinary citrate excretion and reducing urinary calcium excretion through suppressing the expression of transporters of citrate (NADC1) and calcium (CLDN14) in renal tubular epithelial cells (37). Nozawa et al. also found that SCFAs can increase the uptake of organic anions (like formate) in renal HEK293 cells transfected with SLC26A6 (38). However, the underlying mechanisms for the relationship between SCFAs and the expression of oxalate transporter SLC26 are not clear yet.

Based on the existing evidence, we hypothesized that SCFAs derived from gut microbiota may reduce urinary oxalate level and thus prevent renal CaOx crystals by regulating the expression of intestinal oxalate transporters SLC26A3/6. We aimed to study the effect of SCFAs on the formation of renal CaOx stones and explore the role of intestinal oxalate transporters SLC26A3/6 in this process.

## RESULTS

### Oral administration of SCFAs reduced renal CaOx crystals and urinary oxalate level.

**FIG 1:**
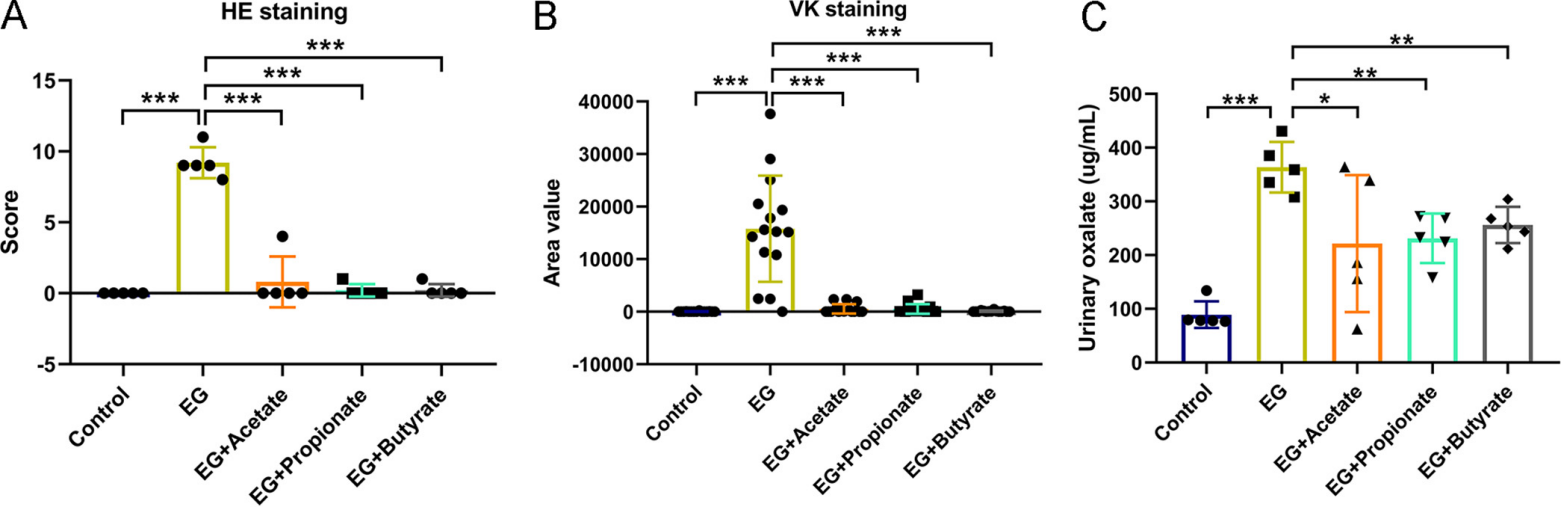
Acetate, propionate, or butyrate reduced renal calcium oxalate crystals and urinary oxalate. (A and B) The HE staining score (A) and VK staining value (B) in five groups: control, ethylene glycol (EG), EG + Acetate, EG + Propionate, and EG + Butyrate. Three sections of each VK staining were captured for calculating area value. (C) The level of oxalate in urine. *n* = 5 rats/group. Student’s *t* test was applied. *P* < 0.05 (*), *P* < 0.01 (**), *P* < 0.001 (***).

We successfully developed renal CaOx stone model rats (ethylene glycol [EG] group) with 1% (vol/vol) EG drinking water. To explore the roles of three main SCFAs produced by gut microbiota (acetic acid, propionic acid, and butyric acid) in reducing renal stones, we provided three subgroups of model rats with sodium acetate (EG + Acetate group), sodium propionate (EG + Propionate group), or sodium butyrate (EG + Butyrate group) in drinking water. As we previously reported, hematoxylin and eosin (HE) staining showed that there were crystals in the lumen of the renal tubules with tubular dilation in model rats, whereas administration of one of the three SCFAs (acetate, propionate, or butyrate) reduced the renal crystals and the abnormality of renal histologic structure (27). Student’s *t* test showed that HE scores (EG versus EG + Acetate: 9.2 versus 0.8, *P* < 0.001; EG versus EG + Propionate: 9.2 versus 0.2, *P* < 0.001; EG versus EG + Butyrate: 9.2 versus 0.2, *P* < 0.001) and area values in Von Kossa (VK) (EG versus EG + Acetate: 15,798 versus 527, *P* < 0.001; EG versus EG + Propionate: 15,798 versus 516, *P* < 0.001; EG versus EG + Butyrate: 15,798 versus 92, *P* < 0.001) decreased sharply in the groups treated with SCFAs compared with the EG group (Fig. 1A and B). The level of urinary oxalate also decreased in rats receiving acetate, propionate, or butyrate (EG versus EG + Acetate: 364 versus 221, *P* = 0.047; EG versus EG + Propionate: 364 versus 231, *P* = 0.002; EG versus EG + Butyrate: 364 versus 256, *P* = 0.003) (Fig. 1C). These data indicated that SCFA treatment significantly reduced urinary oxalate levels and renal CaOx crystals.

### Oral administration of SCFAs regulated expression of the intestinal oxalate transporters SLC26A3/6.

**FIG 2:**
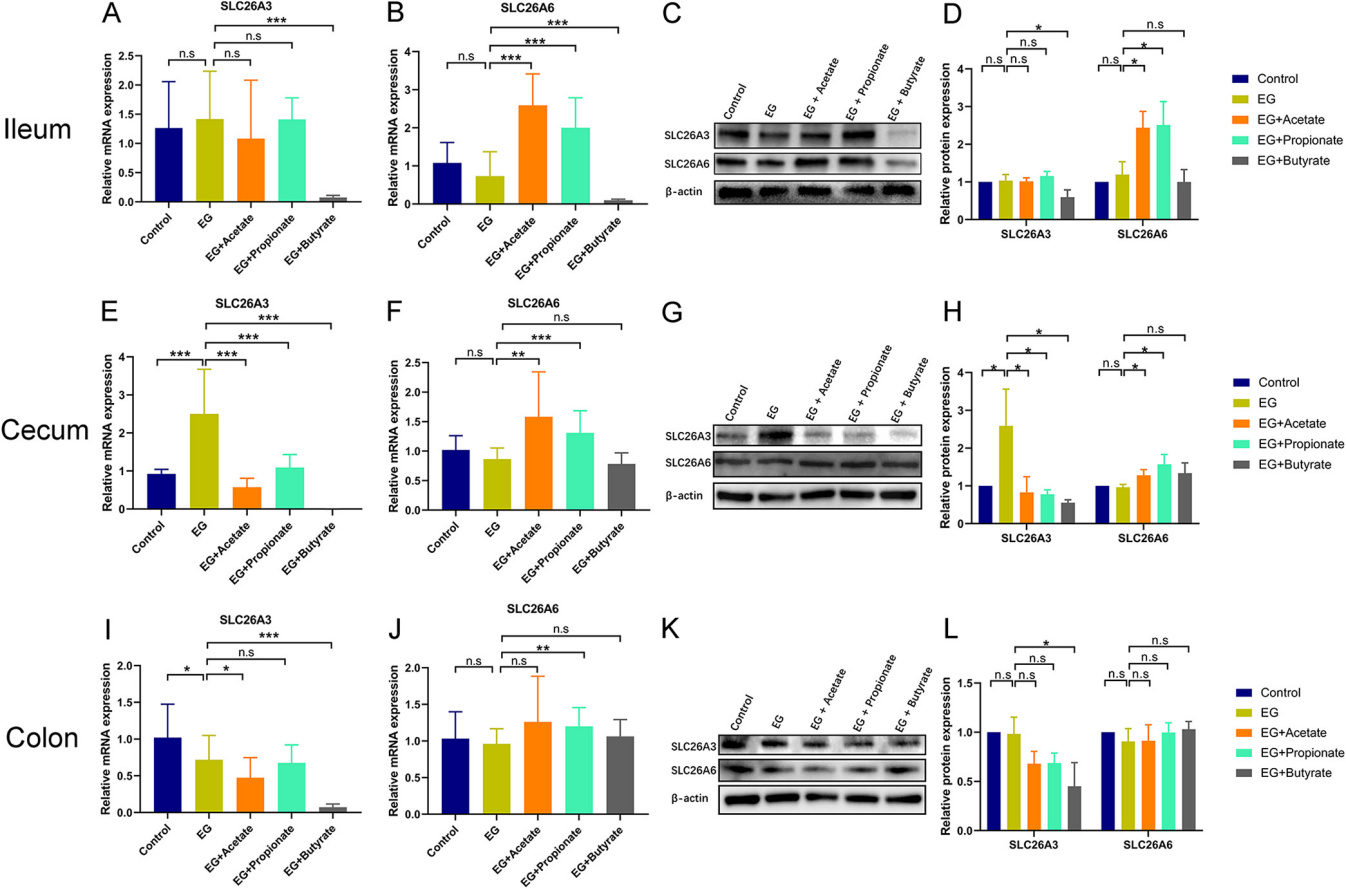
SCFAs regulated the expression of oxalate transporters SLC26A3 and SLC26A6 in the gut. (A to D) The expression of oxalate transporters SLC26A3 and SLC26A6 at mRNA (A and B) and protein (C and D) level in the ileum of rats in control, ethylene glycol (EG), EG + Acetate, EG + Propionate, and EG + Butyrate groups. (E to H) The expression of oxalate transporters SLC26A3 and SLC26A6 at mRNA (E and F) and protein (G and H) level in the cecum. (I to L) The expression of oxalate transporters SLC26A3 and SLC26A6 at mRNA (I and J) and protein (K and L) level in the colon. At mRNA level, five rats of each group were examined, and the duplication was set as 3. At protein level, three rats of each group were examined. The relative protein expression of the control group was set as 1. Student’s *t* test was applied. *P* > 0.05 (not significant [n.s.]), *P* < 0.05 (*), *P* < 0.01 (**), *P* < 0.001 (***).

We next examined whether the decreased urinary oxalate level was associated with the level of intestinal oxalate transporters SLC26A3/6 at mRNA and protein levels using quantitative real-time PCR (qRT-PCR) assay and Western blot assay by Student’s *t* test. The expression of SLC26A3 in the cecum at mRNA (2.7-fold, *P* < 0.001) and protein (2.6-fold, *P* = 0.047) levels was higher in the EG group than the control group (Fig. 2E, G, and H), whereas the level of SLC26A3 decreased in the ileum at mRNA (0.1-fold, *P* < 0.001) and protein (0.6-fold, *P* = 0.038) levels (Fig. 2A, C, and D) and in the colon at mRNA (0.1-fold, *P* < 0.001) and protein (0.5-fold, *P* = 0.035) levels (Fig. 2I, K, and L) after the administration of butyrate and in the cecum after the administration of acetate (mRNA level: 0.2-fold, *P* < 0.001; protein level: 0.3-fold, *P* = 0.045), propionate (mRNA level: 0.4-fold, *P* < 0.001; protein level: 0.3-fold, *P* = 0.033), or butyrate (mRNA level: 0.1-fold, *P* < 0.001; protein level: 0.2-fold, *P* = 0.022) (Fig. 2E, G, and H). The expression of SLC26A6 at mRNA and protein levels was not different between the control group and the EG group (*P* > 0.05). Acetate increased the expression of SLC26A6 in the ileum (mRNA level: 3.5-fold, *P* < 0.001; protein level: 2.1-fold, *P* = 0.017) and cecum (mRNA level: 1.8-fold, *P* = 0.001; protein level: 1.3-fold, *P* = 0.030) (Fig. 2B to D and F to H). The expression of SLC26A6 in the ileum (mRNA level: 2.7-fold, *P* < 0.001; protein level: 2.1-fold, *P* = 0.033) and cecum (mRNA level: 1.5-fold, *P* < 0.001; protein level: 1.6-fold, *P* = 0.044) also increased after the administration of propionate (Fig. 2B to D and F to H). Immunofluorescence assay also showed that butyrate (0.6-fold, *P* = 0.032) decreased intestinal SLC26A3 protein expression, while acetate (2.0-fold, *P* < 0.001) and propionate (1.9-fold, *P* < 0.001) increased intestinal SLC26A6 protein expression in rats (see Fig. S1 in the supplemental material). These data showed that administration of SCFAs could upregulate SLC26A6 and downregulate SLC26A3 expression in the intestine.

10.1128/mSystems.01045-21.1 FIG S1 Acetate, propionate, or butyrate regulated the expression of oxalate transporters SLC26A3 and SLC26A6 in the gut of rats. (A) Immunofluorescence staining (×50) of SLC26A3 protein in the ileum. (B) The immunofluorescence intensity of SLC26A3 protein in the ileum. (C) Immunofluorescence staining (×50) of SLC26A6 protein in the ileum. (D) The immunofluorescence intensity of SLC26A6 protein in the ileum. *n* = 5 rats/group. Student’s *t* test was applied. *P* < 0.05 (*), *P* < 0.001 (***). Download FIG S1, TIF file, 2.9 MB. Copyright © 2021 Liu et al. 2021 Liu et al. https://creativecommons.org/licenses/by/4.0/ This content is distributed under the terms of the Creative Commons Attribution 4.0 International license.

### Oral administration of SCFAs changed gut microbiota and SCFA levels in the content of cecum.

**FIG 3:**
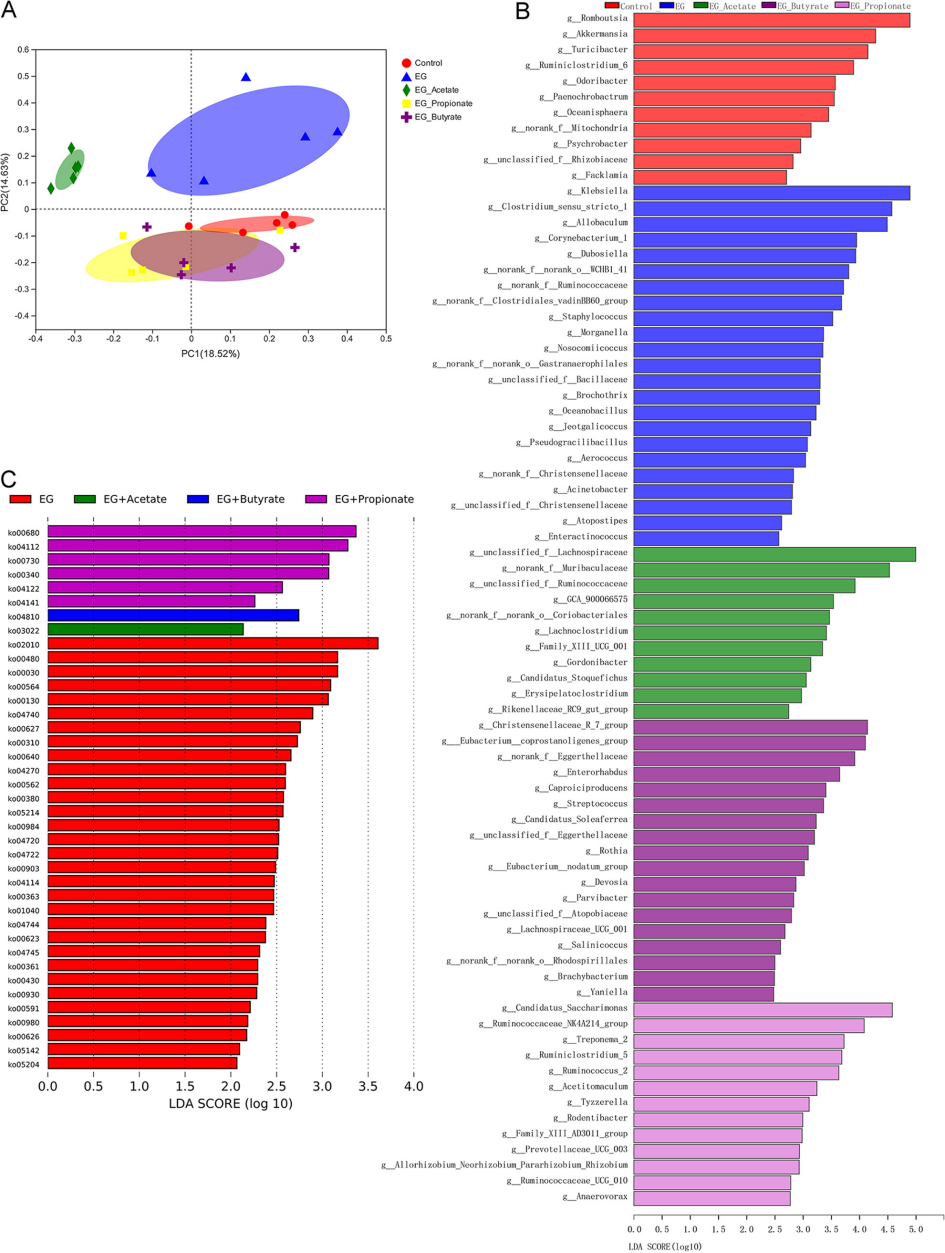
Acetate, propionate, or butyrate regulated the gut microbiota. (A) Beta diversity analysis of gut microbiota in control, ethylene glycol (EG), EG + Acetate, EG + Propionate, and EG + Butyrate groups. (B) Bacteria with higher relative abundance in the five groups of rats. (C) Overrepresented metabolic pathways in the five groups. *n* = 5 rats/group.

After the administration of SCFAs, we also examined the gut microbiota using 16S rRNA gene sequencing. Beta diversity analysis evaluated by principal-coordinate analysis (PCoA) (Bray-Curtis) showed the apparent variation of the gut microbiota from the above five groups, of which the samples could be classified into five clusters (Fig. 3A, analysis of similarity [ANOSIM], *R* = 0.365, *P* = 0.001; ADONIS test, *R*^2^ = 0.272, *P* = 0.001). Linear discriminant analysis effect size (LEfSe) analysis showed that the relative abundances of *Lachnospiraceae* (*P* = 0.010), *Ruminococcus* (*P* = 0.006), *Eubacterium* (*P* = 0.003), and *Prevotellaceae* (*P* = 0.028), which can produce SCFAs, were higher in rats receiving acetate, propionate, or butyrate (Fig. 3B). Moreover, the administration of propionate also increased the abundance of *O. formigenes* (*P* = 0.007) (Fig. S2). We obtained metabolic pathway information using Tax4Fun. Metabolic pathway analysis based on the Kyoto Encyclopedia of Genes and Genomes (KEGG) database showed that propionate increased the pathway ko00680 (methane metabolism), which is involved in the production of acetic acid (*P* = 0.017) (Fig. 3C).

10.1128/mSystems.01045-21.2 FIG S2 Bacteria with higher relative abundance in the gut microbiota of ethylene glycol (EG) and EG + Propionate groups. Download FIG S2, TIF file, 0.5 MB. Copyright © 2021 Liu et al. 2021 Liu et al. https://creativecommons.org/licenses/by/4.0/ This content is distributed under the terms of the Creative Commons Attribution 4.0 International license.

**FIG 4:**
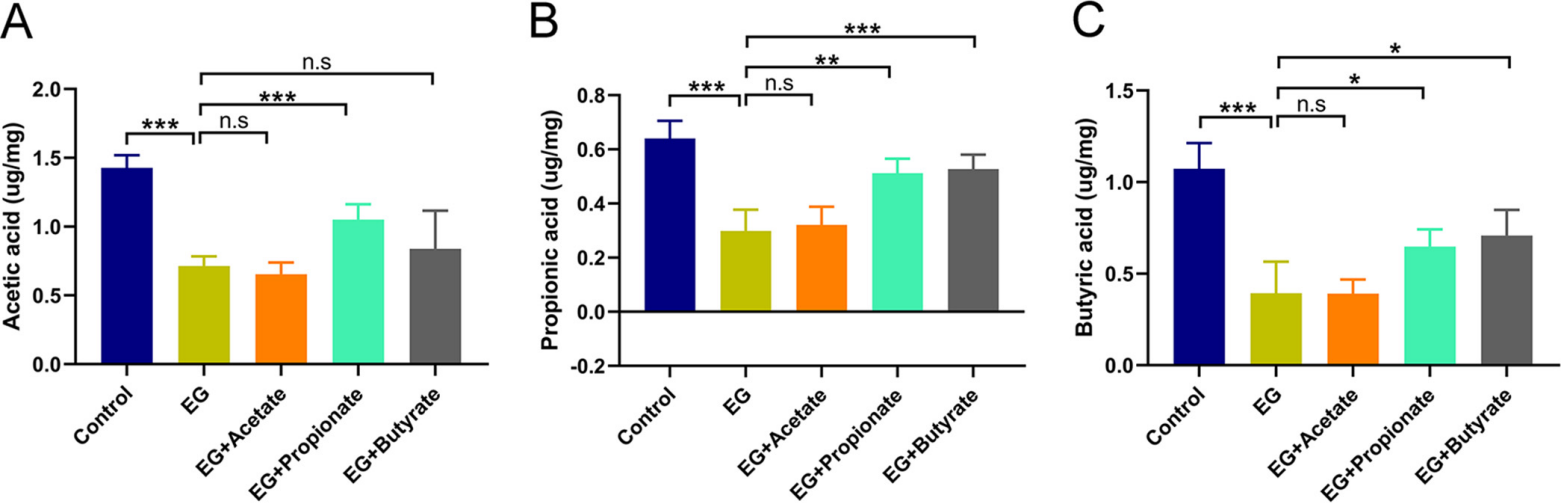
Acetate, propionate, or butyrate regulated the level of acetic acid (A), propionic acid (B), and butyric acid (C) in the content of cecum from the five groups. *n* = 5 rats/group. Student’s *t* test was applied. *P* > 0.05 (n.s.), *P* < 0.05 (*), *P* < 0.01 (**), *P* < 0.001 (***).

Student’s *t* test showed that the level of acetic acid in the content of cecum increased in rats administered propionate compared with model rats (1.05 versus 0.71 μg/mg, *P* < 0.001) (Fig. 4A). Both cecal propionic acid (EG + Propionate versus EG: 0.51 versus 0.30 μg/mg, *P* = 0.001; EG + Butyrate versus EG: 0.53 versus 0.30 μg/mg, *P* < 0.001) and butyric acid (EG + Propionate versus EG: 0.65 versus 0.40 μg/mg, *P* = 0.020; EG + Butyrate versus EG: 0.71 versus 0.40 μg/mg, *P* = 0.013) increased in propionate- and butyrate-supplemented groups over the model group (Fig. 4B and C). These results suggested that administration of SCFAs also changed the gut microbiota and SCFA levels in the content of cecum.

### Depletion of gut microbiota increased CaOx crystal deposition in kidneys.

**FIG 5:**
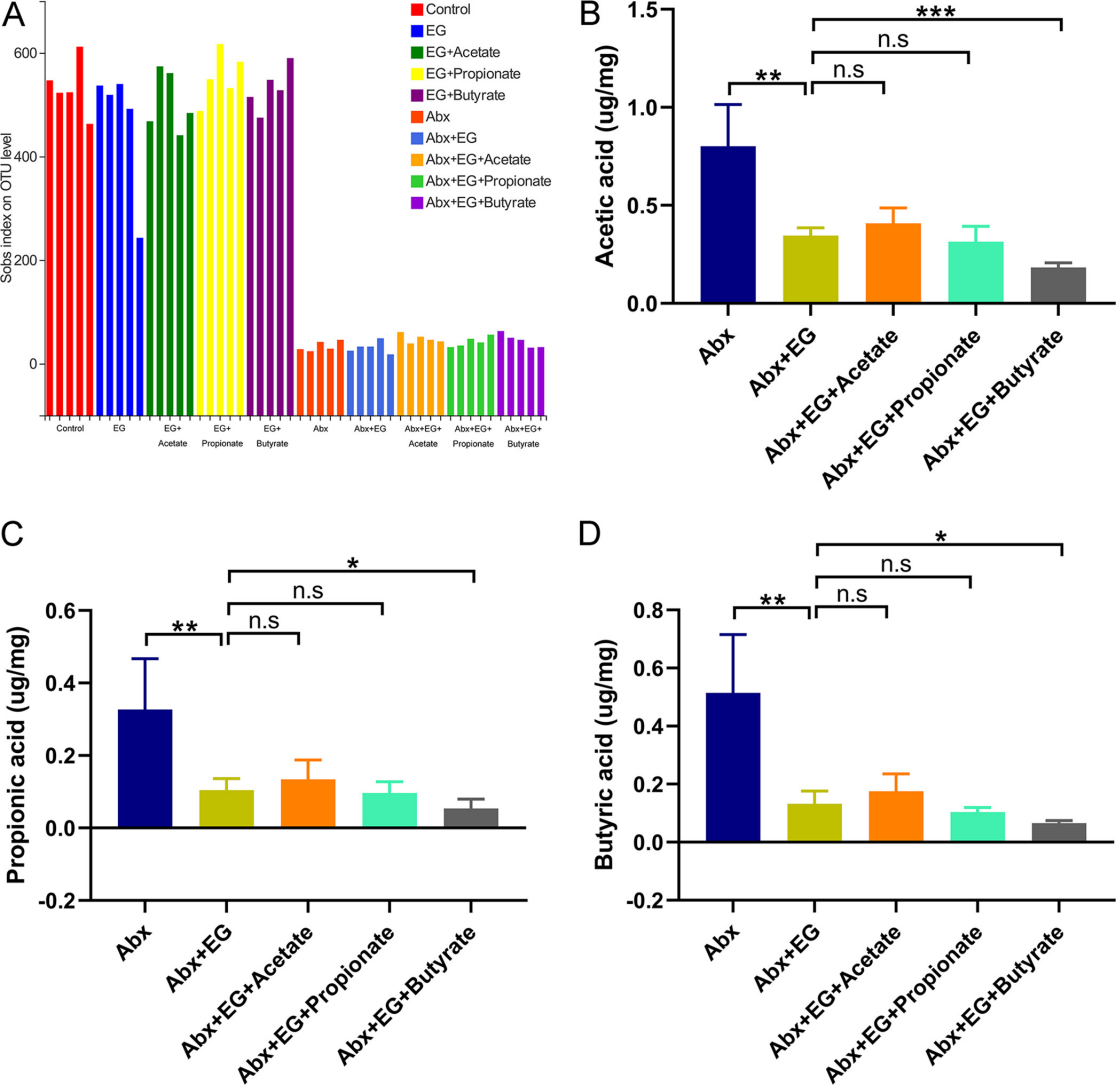
The SCFA levels of contents in the cecum did not increase in rats receiving acetate, propionate, or butyrate after depletion of gut microbiota using antibiotics. (A) Alpha diversity of gut microbiota in the rats of control, ethylene glycol (EG), EG + Acetate, EG + Propionate, and EG + Butyrate groups with or without antibiotics. (B to D) The level of acetic acid (B), propionic acid (C), and butyric acid (D) in the contents of the cecum of rats in antibiotics (Abx), Abx + EG, Abx + EG + Acetate, Abx + EG + Propionate, and Abx + EG + Butyrate groups. *n* = 5 rats/group. Student’s *t* test was applied. *P* > 0.05 (n.s.), *P* < 0.05 (*), *P* < 0.01 (**), *P* < 0.001 (***).

**FIG 6:**
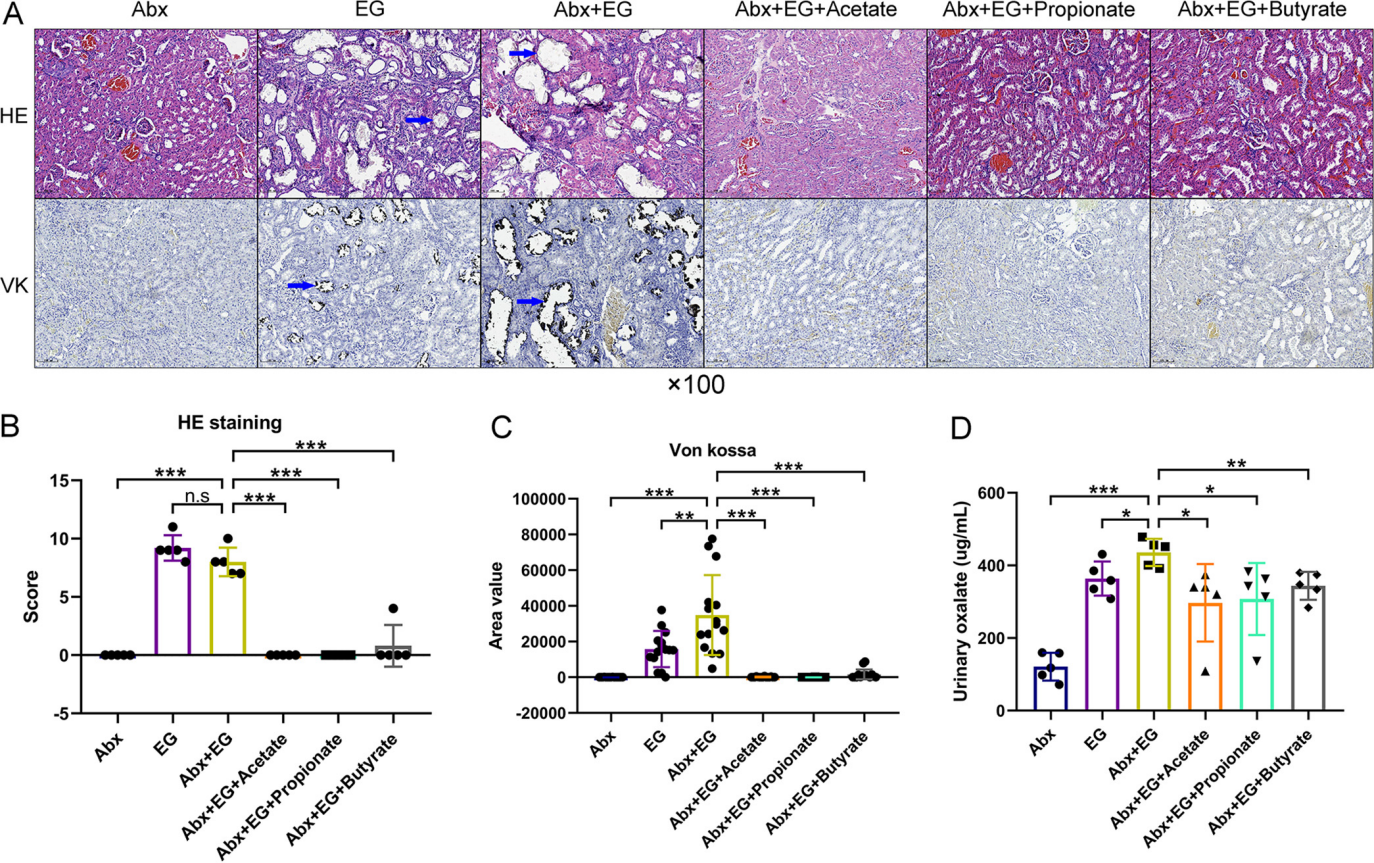
Depletion of gut microbiota by antibiotics increased renal calcium oxalate crystals and urinary oxalate levels, which were again alleviated by the administration of acetate, propionate, or butyrate in rats. (A) Hematoxylin and eosin (HE, ×100) and Von Kossa (VK, ×100) staining of renal tissues of rats in antibiotics (Abx), ethylene glycol (EG), Abx + EG, Abx + EG + Acetate, Abx + EG + Propionate, and Abx + EG + Butyrate groups. Blue arrows show calcium oxalate crystals. (B and C) The HE staining score (B) and VK staining value (C). Three sections of each VK staining were captured for calculating area value). (D) The level of oxalate in urine. *n* = 5 rats/group. Student’s *t* test was applied. *P* > 0.05 (n.s.), *P* < 0.05 (*), *P* < 0.01 (**), *P* < 0.001 (***).

To determine whether gut microbiota also regulated the formation of renal stones, we used antibiotics (Abx) to reduce the intestinal bacterial load and examined the change of crystal deposition in kidneys. Alpha diversity analysis using the sobs index showed that the administration of antibiotics significantly decreased the richness of gut microbiota in model rats (Fig. 5A). Student’s *t* test showed that model rats with antibiotics had higher VK staining scores than those without antibiotics (34,851 versus 15,789, *P* = 0.006) (Fig. 6A and C). The urinary oxalate level was also higher in model rats with antibiotics than those without (436 versus 364 μg/ml, *P* = 0.028) (Fig. 6D). These results suggested that the depletion of gut microbiota could promote the urinary oxalate level and the formation of renal crystals.

### Supplementation of SCFAs with depletion of gut microbiota decreased renal crystals via regulating expression of intestinal oxalate transporters SLC26A3/6.

To explore whether SCFAs decreased the renal stone formation via the gut microbiota, the gut microbiota of these five groups were depleted by antibiotics. The alpha diversity (sobs index) of gut microbiota of these five groups with antibiotics was much lower than that of those without antibiotics (*P* < 0.05) (Fig. 5A). The SCFAs in the content of cecum did not increase after administration of acetate, propionate, or butyrate (Fig. 5B to D).

The renal crystals and renal tubular dilation also decreased after the administration of acetate, propionate, or butyrate with antibiotics (Fig. 6A). Student’s *t* test showed that the HE scores (Abx + EG versus Abx + EG + Acetate: 8.0 versus 0, *P* < 0.001; Abx + EG versus Abx + EG + Propionate: 8.0 versus 0, *P* < 0.001; Abx + EG versus Abx + EG + Butyrate: 8.0 versus 0.8, *P* < 0.001), area values in VK staining (Abx + EG versus Abx + EG + Acetate: 34,851 versus 130, *P* < 0.001; Abx + EG versus Abx + EG + Propionate: 34,851 versus 41, *P* < 0.001; Abx + EG versus Abx + EG + Butyrate: 34,851 versus 1,592, *P* < 0.001) (Fig. 6B and C), and level of urinary oxalate (Abx + EG versus Abx + EG + Acetate: 436 versus 297, *P* = 0.025; Abx + EG versus Abx + EG + Propionate: 436 versus 308, *P* = 0.027; Abx + EG versus Abx + EG + Butyrate: 436 versus 344, *P* = 0.005) (Fig. 6D) decreased in rats receiving acetate, propionate, or butyrate after depletion of gut microbiota compared with rats in the Abx + EG group.

**FIG 7:**
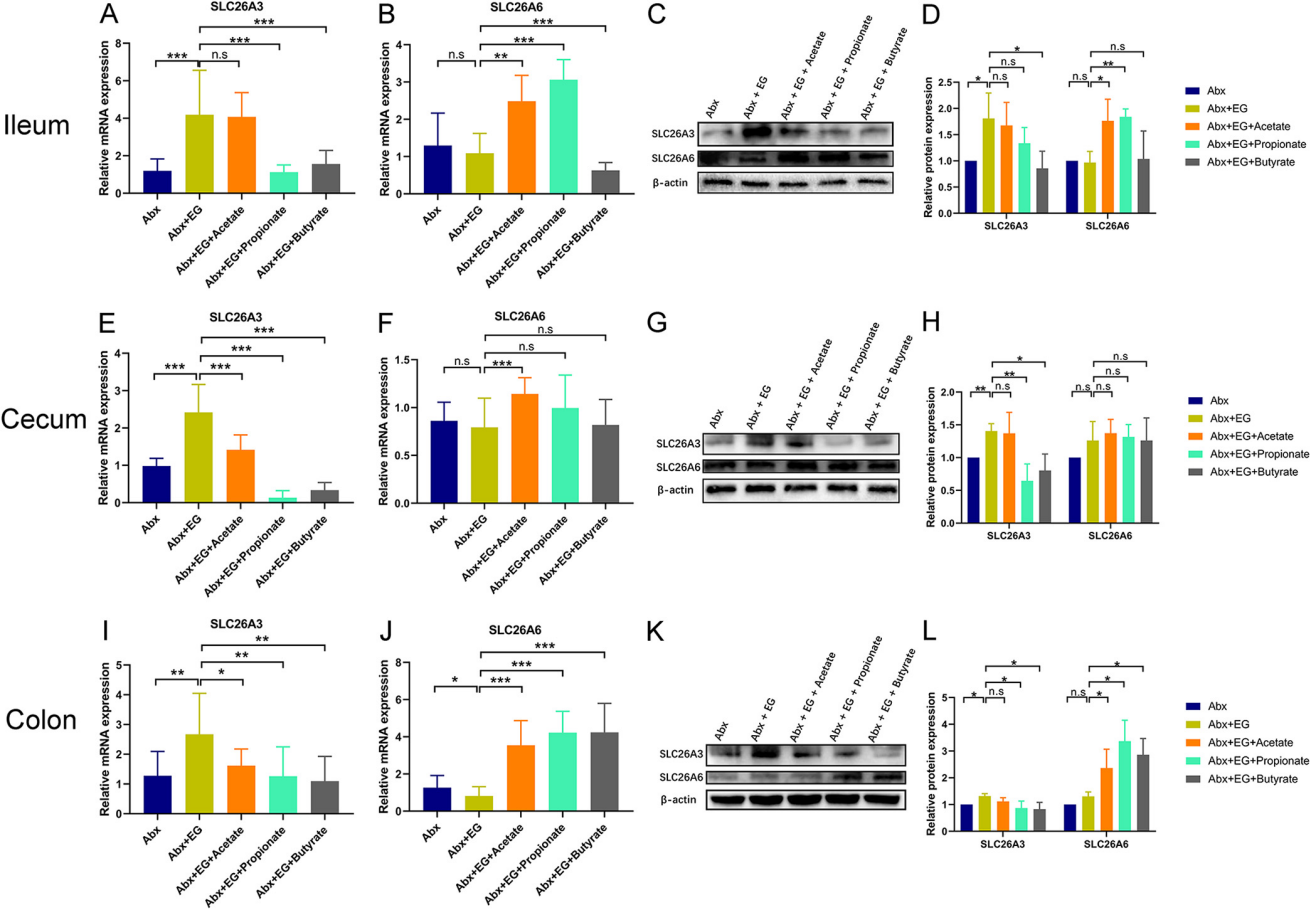
Acetate, propionate, or butyrate regulated the expression of oxalate transporters SLC26A3 and SLC26A6 in the gut of rats receiving antibiotics. (A to D) The expression of oxalate transporters SLC26A3 and SLC26A6 at mRNA (A and B) and protein (C and D) level in the ileum of rats in antibiotics (Abx), ethylene glycol (EG), Abx + EG, Abx + EG + Acetate, Abx + EG + Propionate, and Abx + EG + Butyrate groups. (E to H) The expression of oxalate transporters SLC26A3 and SLC26A6 at mRNA (E and F) and protein (G and H) level in the cecum. (I to L) The expression of oxalate transporters SLC26A3 and SLC26A6 at mRNA (I and J) and protein (K and L) level in colon. At mRNA level, five rats of each group were examined, and the duplication was set as 3. At protein level, three rats of each group were examined. The relative protein expression of control group was set as 1. Student’s *t* test was applied. *P* > 0.05 (n.s.), *P* < 0.05 (*), *P* < 0.01 (**), *P* < 0.001 (***).

The expression of intestinal oxalate transporters SLC26A3/6 was also measured at mRNA and protein levels using qRT-PCR assay and Western blot assay by Student’s *t* test. After antibiotic treatment, the expression of SLC26A3 in the ileum (mRNA level: 3.5-fold, *P* < 0.001; protein level: 1.8-fold, *P* = 0.045) (Fig. 7A, C, and D), cecum (mRNA level: 2.5-fold, *P* < 0.001; protein level: 1.4-fold, *P* = 0.003) (Fig. 7E, G, and H), and colon (mRNA level: 2.1-fold, *P* = 0.007; protein level: 1.3-fold, *P* = 0.004) (Fig. 7I, K, and L) was higher in the Abx + EG group than the Abx group. Meanwhile, the administration of propionate significantly decreased the expression of SLC26A3 compared with the Abx + EG group in the cecum (mRNA level: 0.1-fold, *P* < 0.001; protein level: 0.5-fold, *P* = 0.009) (Fig. 7E, G, and H) and colon (mRNA level: 0.5-fold, *P* = 0.008; protein level: 0.7-fold, *P* = 0.049) (Fig. 7I, K, and L). Butyrate could also reduce SLC26A3 expression in the ileum (mRNA level: 0.4-fold, *P* < 0.001; protein level: 0.5-fold, *P* = 0.049) (Fig. 7A, C, and D), cecum (mRNA level: 0.1-fold, *P* < 0.001; protein level: 0.6-fold, *P* = 0.019) (Fig. 7E, G, and H), and colon (mRNA level: 0.4-fold, *P* = 0.002; protein level: 0.6-fold, *P* = 0.035) (Fig. 7I, K, and L). The expression of SLC26A6 increased in the ileum after administration of acetate (mRNA level: 2.3-fold, *P* = 0.001; protein level: 1.8-fold, *P* = 0.041) or propionate (mRNA level: 2.8-fold, *P* < 0.001; protein level: 1.9-fold, *P* = 0.005) with antibiotics (Fig. 7B to D), and it also increased in the colon after the administration of acetate (mRNA level: 4.4-fold, *P* < 0.001; protein level: 1.8-fold, *P* = 0.045), propionate (mRNA level: 5.2-fold, *P* < 0.001; protein level: 2.6-fold, *P* = 0.011), or butyrate (mRNA level: 5.2-fold, *P* < 0.001; protein level: 2.2-fold, *P* = 0.013) (Fig. 7J to L) with antibiotic treatment. These results suggested that extra administration of acetate, propionate, or butyrate could directly reduce renal CaOx stones and urinary oxalate level in rats with antibiotic-depleted gut microbiota and regulate the levels of oxalate transporters SLC26A3/6 in the intestine.

### SCFAs changed the expression of SLC26A3/6 in Caco-2 cells.

**FIG 8:**
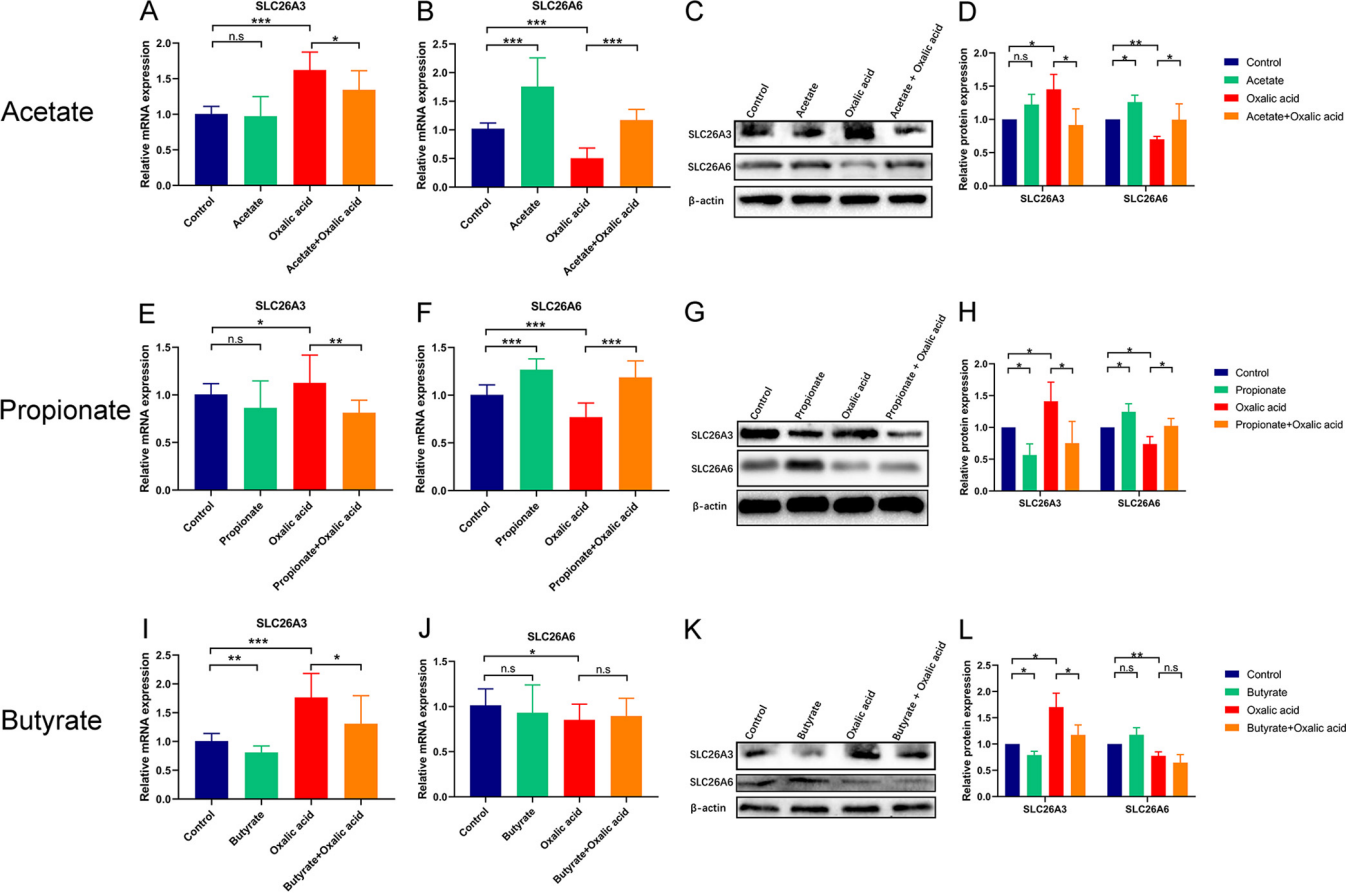
Propionate and butyrate decreased oxalate transporter SLC26A3 expression, while acetate and propionate increased oxalate transporter SLC26A3 expression in human epithelial cell line Caco-2. (A to D) The effect of oxalic acid and/or acetate on the expression of oxalate transporters SLC26A3 and SLC26A6 at mRNA (A and B) and protein (C and D) level. (E to H) The effect of oxalic acid and/or propionate on the expression of oxalate transporters SLC26A3 and SLC26A6 at mRNA (E and F) and protein (G and H) level. (I to L) The effect of oxalic acid and/or butyrate on the expression of oxalate transporters SLC26A3 and SLC26A6 at mRNA (I and J) and protein (K and L) level. The relative protein expression of the control group was set as 1. Each experiment was repeated three times. The duplication was set as 3 at mRNA level. Student’s *t* test was applied. *P* > 0.05 (n.s.), *P* < 0.05 (*), *P* < 0.01 (**), *P* < 0.001 (***).

To examine the direct effect of SCFAs on the expression of SLC26A3/6 in intestinal cells, human intestinal epithelial Caco-2 cells were used as a model and were treated with sodium acetate, sodium propionate, or sodium butyrate. We first used different concentrations of SCFAs (0, 0.1, 1, 5, 10, and 20 mM) and oxalic acid (0, 0.01, 0.05, 0.1, 0.5, and 1 mM) to stimulate Caco-2 cells (Fig. S3). Then, we selected 5 mM acetate, 5 mM propionate, 1 mM butyrate, and/or 0.1 mM oxalic acid to treat Caco-2 cells since the significant changes of SLC26A3/6 at mRNA level were observed at these concentrations using Student’s *t* test. The protein expression of SLC26A3 increased in Caco-2 cells treated with oxalic acid (Fig. 8A, C to E, G to I, K, and L). Acetate (mRNA level: 0.8-fold, *P* = 0.043; protein level: 0.6-fold, *P* = 0.048) (Fig. 8A, C, and D), propionate (mRNA level: 0.7-fold, *P* = 0.009; protein level: 0.6-fold, *P* = 0.013) (Fig. 8E, G, and H), and butyrate (mRNA level: 0.7-fold, *P* = 0.022; protein level: 0.8-fold, *P* = 0.007) (Fig. 8I, K, and L) could inhibit the protein expression of SLC26A3. On the other hand, the level of SLC26A6 protein in Caco-2 cells decreased once exposed to oxalic acid (Fig. 8B to D, F to H, and J to L) and increased after the treatment with acetate (mRNA level: 2.3-fold, *P* < 0.001; protein level: 1.3-fold, *P* = 0.013) (Fig. 8B to D) or propionate (mRNA level: 1.5-fold, *P* < 0.001; protein level: 1.2-fold, *P* = 0.027) (Fig. 8F to H). The results indicated that SCFAs could directly affect the expression of oxalate transporters SLC26A3/6 in intestinal cells.

10.1128/mSystems.01045-21.3 FIG S3 Oxalic acid, acetate, propionate, and butyrate regulated the expression of oxalate transporters SLC26A3 and SLC26A6 at mRNA level. (a and b) The effect of oxalic acid on the expression of SLC26A3 (a) and SLC26A6 (b). (c and d) The effect of acetate on the expression of SLC26A3 (c) and SLC26A6 (d). (e and f) The effect of propionate on the expression of SLC26A3 (e) and SLC26A6 (f). (g and h) The effect of butyrate on the expression of SLC26A3 (g) and SLC26A6 (h). Each experiment was repeated three times. The duplication was set as 3. *P* < 0.05 (*), *P* < 0.01 (**), *P* < 0.001 (***). Download FIG S3, TIF file, 1.4 MB. Copyright © 2021 Liu et al. 2021 Liu et al. https://creativecommons.org/licenses/by/4.0/ This content is distributed under the terms of the Creative Commons Attribution 4.0 International license.

### The protective effect of SCFAs against renal CaOx stones was attenuated by suppression of intestinal SLC26A6 but not by overexpression of intestinal SLC26A3.

**FIG 9:**
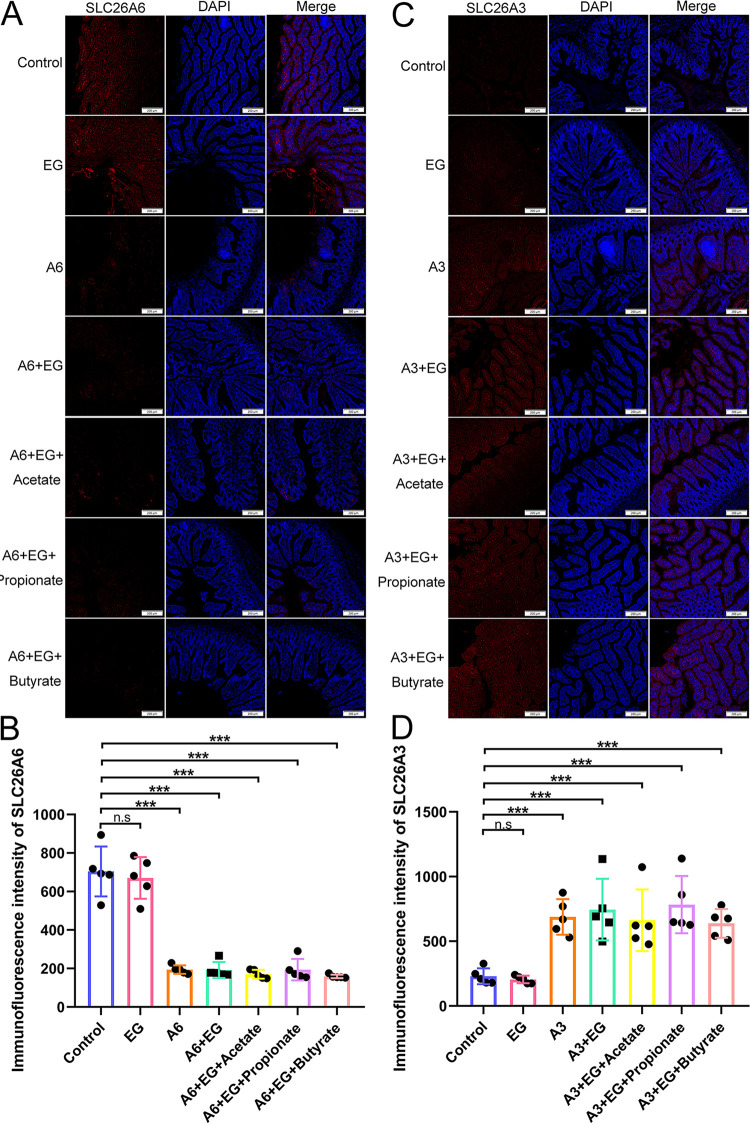
The expression of SLC26A6 protein decreased in the intestine after receiving adeno-associated virus 9 (AAV9)-SLC26A6-shRNA, while that of SLC26A3 protein increased in the intestine after receiving AAV9-SLC26A3. (A) Immunofluorescence staining (×50) of SLC26A6 protein in the intestine. (B) The immunofluorescence intensity of SLC26A6 protein in the intestine. (C) Immunofluorescence staining (×50) of SLC26A3 protein in the intestine. (D) The immunofluorescence intensity of SLC26A3 protein in the intestine. *n* = 5 rats/group. Student’s *t* test was applied. *P* > 0.05 (n.s.), *P* < 0.001 (***).

To verify the role of intestinal oxalate transporters SLC26A3 and SLC26A6 in the reduction of renal CaOx crystals induced by SCFAs, SLC26A3 was overexpressed by adeno-associated virus 9 (AAV9)-SLC26A3, and SLC26A6 was suppressed by AAV9-SLC26A6-shRNA (short hairpin RNA) in rats. The qRT-PCR assay and immunofluorescence assay showed that the expression of intestinal SLC26A6 at mRNA (Fig. S4A) and protein (Fig. 9A and B) levels decreased significantly in rats receiving AAV9-SLC26A6-shRNA (*P* < 0.001). AAV9-SLC26A3 contributed to the significant increase of the expression of intestinal SLC26A3 at mRNA (Fig. S4B) and protein (Fig. 9C and D) levels (*P* < 0.001).

10.1128/mSystems.01045-21.4 FIG S4 The effect of adeno-associated virus 9 (AAV9)-SLC26A6-shRNA and AAV9-SLC26A3 on the expression of SLC26A3/6 at mRNA level in the intestine of rats. (A) The expression of SLC26A6 at mRNA level decreased in the intestine after receiving AAV9-SLC26A6-shRNA. (B) The expression of SLC26A3 at mRNA level increased in the intestine after receiving AAV9-SLC26A3. Student’s *t* test was applied. *P* > 0.05 (n.s.), *P* < 0.001 (***). Download FIG S4, TIF file, 0.2 MB. Copyright © 2021 Liu et al. 2021 Liu et al. https://creativecommons.org/licenses/by/4.0/ This content is distributed under the terms of the Creative Commons Attribution 4.0 International license.

**FIG 10:**
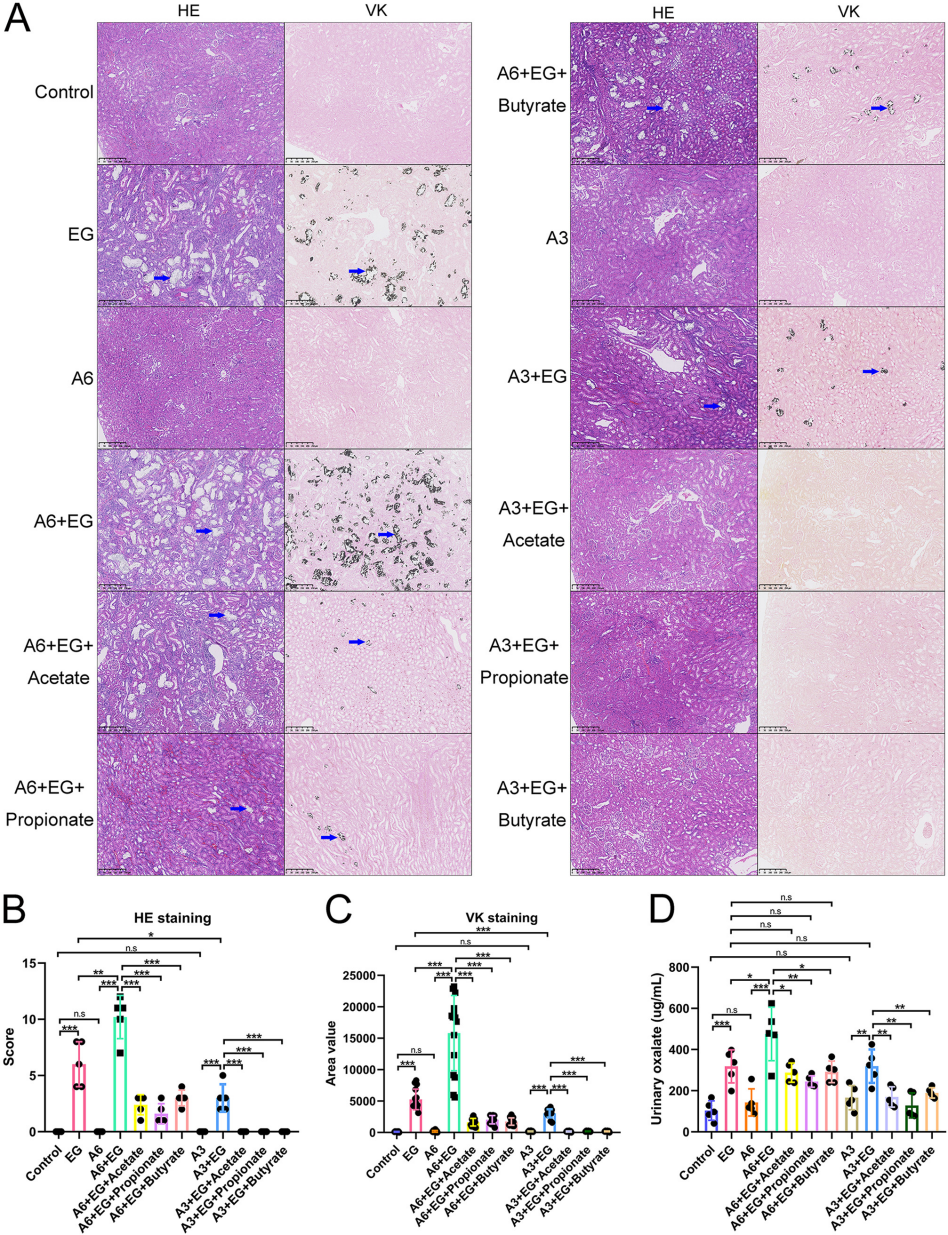
The decrease of intestinal SLC26A6 expression increased renal crystals, while the increase of intestinal SLC26A3 expression decreased renal crystals. (A) Hematoxylin and eosin (HE, ×100) and Von Kossa (VK, ×100) staining of renal tissues of rats in control, ethylene glycol (EG), A6, A6+EG, A6+EG+Acetate, A6+EG+Propionate, A6+EG+Butyrate, A3, A3+EG, A3+EG+Acetate, A3+EG+Propionate, and A3+EG+Butyrate groups. Blue arrows show calcium oxalate crystals. (B and C) The HE staining score (B) and VK staining value (C) in 12 groups. Three sections of each VK staining were captured for calculating area value. (D) The level of oxalate in urine. *n* = 5 rats/group. Student’s *t* test was applied. *P* > 0.05 (n.s.), *P* < 0.05 (*), *P* < 0.01 (**), *P* < 0.001 (***).

HE and VK staining showed that the decrease of SLC26A6 expression or the increase of SLC26A3 expression in the intestine alone did not increase the amount of CaOx crystals in kidney nor the level of urinary oxalate (*P* > 0.05) with Student’s *t* test. However, after the administration of EG, the decrease of intestinal SLC26A6 expression contributed to more renal crystals (HE scores: 6 versus 10.2, *P* = 0.010; area values in VK staining: 5,266.73 versus 15,804.3, *P* < 0.001) and higher urinary oxalate level (318 versus 476 μg/ml, *P* = 0.049). In contrast to the results mentioned above, renal crystals also existed after the decrease of intestinal SLC26A6 expression even though acetate, propionate, or butyrate was provided. In addition, the urinary oxalate levels between EG group (318 μg/ml) and A6+EG+Acetate group (288 μg/ml), A6+EG+Propionate group (245 μg/ml), or A6+EG+Butyrate group (292 μg/ml) were not significantly different (*P* > 0.05). Interestingly, the increase of intestinal SLC26A3 expression decreased the renal crystals (HE scores: 6 versus 3, *P* = 0.021; area values in VK staining: 5,266.73 versus 3,041.73, *P* < 0.001). However, the urinary oxalate levels were not significantly different between EG group and EG+A3 group (318 versus 319 μg/ml, *P* > 0.05). There was no crystal in the kidney in the A3+EG+Acetate, A3+EG+Propionate, and A3+EG+Butyrate groups. The results of HE and VK staining and urinary oxalate level were shown in Fig. 10. These results revealed that the intestinal SLC26A6 played a major role in the protective effect of SCFAs against renal CaOx stones. Intestinal SLC26A3 also participated in the formation of renal crystals but not by regulating the oxalate metabolism.

## DISCUSSION

Recently, several studies have demonstrated that renal stones may be associated with gut microbiota (9, 10, 39, 40). Our previous study showed that the abundance of SCFA-producing bacteria was lower in the gut microbiota of patients with renal CaOx stones than in healthy people (41), which indicated a possible preventive role of SCFAs in renal stone formation. In this study, the results showed that SCFAs, including acetate, propionate, and butyrate, could reduce renal CaOx stones and urinary oxalate levels via regulating oxalate transporter (SLC26A3/A6) expression in rats, and the phenomenon also existed after the depletion of gut microbiota. The protective role of SCFAs against renal CaOx stones was attenuated after the decrease of intestinal SLC26A6 expression. Intestinal SLC26A3 also participated in the formation of renal crystals but not by regulating the oxalate metabolism.

SLC26 is a family of anion transporters with 10 different subtypes, of which SLC26A3 and SLC26A6 significantly affected the oxalate transportation in the intestine (16). Many studies have already verified the causal relationship between oxalate transporter SLC26, oxalate metabolism, and renal CaOx crystals. In the past 2 decades, five studies reported that SLC26A6 knockout mice developed renal CaOx stones, hyperkalemia, and hyperoxaluria due to the enhanced net oxalate absorption in the ileum (19, 20, 42–44). Our study also verified that the decrease of intestinal SLC26A6 induced more renal crystals. We also found that acetate or propionate could increase the expression of SLC26A6 in the ileum, while this phenomenon weakened in cecum and colon. It may be due to the varied levels of SLC26A6 expression in different parts of the intestine. SLC26A6 has been demonstrated to have higher levels of expression in the small intestine than in the colon (18).

Another study showed that all segments of the intestine in SLC26A3 knockout mice exhibited net oxalate secretion, and 24-h urinary oxalate level decreased by 66% compared with control mice (21). However, our study found that the increase of intestinal SLC26A3 expression did not increase the urinary oxalate level. In contrast, it reduced renal crystals. It was reported previously that intestinal SLC26A3 was in charge of bicarbonate ion secretion and chloride ion and water absorption and that patients with SLC26A3 mutations would suffer from acidic diarrhea and systemic alkalosis (17). Thus, we hypothesized that the overexpression of SLC26A3 in the intestine may lead to less excretion of bicarbonate ions into urine and decrease renal CaOx crystal formation. On the other hand, SLC26A3 also played an important role in maintaining the intestinal epithelial barrier function (45). The mutation of SLC26A3 was associated with inflammatory bowel disease (IBD) (46). Additionally, IBD patients had a 2-fold-higher risk of urolithiasis than non-IBD individuals (47). Thus, mutation of intestinal SLC26A3 may be a risk factor for renal CaOx stones. These pieces of evidence indicated that the intestinal SLC26A3 also played an important role in preventing, rather than contributing to, the formation of renal CaOx stones.

Gut microbiota was also related to many diseases, including metabolic syndrome, autoimmune diseases, colorectal cancer, and inflammatory bowel disease (48–51). In addition, it was reported that the relative abundance of *O. formigenes* was lower in the gut microbiota of renal stone patients than in healthy people (52). However, the application of probiotics containing *O. formigenes* for decreasing urinary oxalate levels was still controversial (25, 26). Intriguingly, in our study, we found that the administration of propionate could elevate the abundance of *O. formigenes* in model rats with decreased urinary oxalate level. With the development of 16S rRNA gene sequencing, some studies found that apart from *O. formigenes*, there were also some other different gut bacteria between renal stone patients and healthy controls (10, 39, 40). For example, *Bacteroides* are higher in renal stone patients, whereas *Faecalibacterium*, Eubacterium hallii, *Dorea*, *Ruminiclostridium*, and *Fusicatenibacter*, which can produce SCFAs, are higher in healthy people. These studies indicated that the disturbance of the gut microbiota might be associated with the formation of renal stones. In our study, we found that SCFA-producing bacteria increased after the administration of SCFAs. The depletion of gut microbiota by antibiotics promoted the deposition of renal crystals. These pieces of evidence indicated that gut microbiota plays a critical role in preventing renal stones. Nevertheless, we also found that oral supplementation with SCFAs of rats with the gut microbiota depleted could also reduce the risk of renal stones. This study suggested that SCFAs could be used as prebiotics to prevent renal stones even though the gut microbiota disorder existed. Nevertheless, further drug toxicology evaluations and clinical trials are needed before SCFAs can be used as treatments for renal CaOx stones. To this end, further efforts to reveal the details of how SCFAs and the gut microbiome function in renal stone alleviation are required.

In conclusion, SCFAs could reduce urinary oxalate and renal CaOx stones through the oxalate transporter SLC26A6 in the intestine. SCFAs may be new supplements for preventing the formation of renal CaOx stones.

## MATERIALS AND METHODS

### Renal stone model and treatment regimes.

Rats (Sprague-Dawley rats, 6 weeks old, male), obtained from Chengdu Dossy Experimental Animals Co., Ltd. (Chengdu, Sichuan, China), were acclimatized for 1 week before the experiment, followed by a 4-week cohousing period in the specific-pathogen-free animal facility at the Animal Experiment Center of West China Hospital, Sichuan University. All rats were singly housed and provided with sterilized food and water. The West China Hospital of Sichuan University Medical Research Ethics Committee approved the study (2017063A).

First, 25 rats were equally divided into five groups, named control group, model group (EG), acetate group (EG + Acetate), propionate group (EG + Propionate), and butyrate group (EG + Butyrate). Rats in the control group got free access to sterile tap water as drinking water. We added 1% (vol/vol) EG into the drinking water to build renal CaOx stone rats. Acetate, propionate, or butyrate groups received drinking water with 1% (vol/vol) EG and 150 mM sodium acetate, sodium propionate, or sodium butyrate, respectively. Second, another 25 rats were also equally divided into five groups. All rats received the same treatment mentioned above. Based on it, we added extra 4 different Abx into drinking water 2 weeks before the administration of SCFAs and throughout the entire experiment, including vancomycin (0.25 mg/ml), metronidazole (0.5 mg/ml), ampicillin (0.5 mg/ml), and neomycin (0.5 mg/ml) (53). The names of these five groups were Abx, Abx + EG, Abx + EG + Acetate, Abx + EG + Propionate, and Abx + EG + Butyrate. Finally, based on the first part of the experiment, 60 rats were equally divided into 12 groups for SLC26A6 suppression and SLC26A3 overexpression experiments. We used AAV9-SLC26A6-shRNA and AAV9-SLC26A3 provided by Shanghai Genechem Co., Ltd. Apart from the control group and EG group, five groups of rats received AAV9-SLC26A6-shRNA (A6 group), AAV9-SLC26A6-shRNA+EG (A6+EG group), AAV9-SLC26A6-shRNA+EG+Acetate (A6+EG+Acetate group), AAV9-SLC26A6-shRNA+EG+Propionate (A6+EG+Propionate group), and AAV9-SLC26A6-shRNA+EG+Butyrate (A6+EG+Butyrate group). Another five groups of rats received AAV9-SLC26A3 (A3 group), AAV9-SLC26A3+EG (A3+EG group), AAV9-SLC26A3+EG+Acetate (A3+EG+Acetate group), AAV9-SLC26A3+EG+Propionate (A3+EG+Propionate group), and AAV9-SLC26A3+EG+Butyrate (A3+EG+Butyrate group). Each rat was injected with 8 × 10^11^ vector genome plasmids through the caudal vein 4 weeks before the supplementation with EG, acetate, propionate, and butyrate.

After 4 weeks, we collected 24-h urine and blood plasma. Renal and intestinal (ileum, cecum, and colon) tissues were fixed in 10% formaldehyde and embedded in paraffin. The remaining parts of these tissues, the corresponding intestinal content, and urine and blood plasma samples were all stored at −80°C for future analyses.

### Cell culture experiments.

The human intestinal epithelial line Caco-2 is a kind of human colon adenocarcinoma cell line, which has a similar structure and function as intestinal epithelial cells. Caco-2 cells were cultured in 1640 medium (HyClone, Logan, UT, USA) with 10% fetal bovine serum (Gibco, Carlsbad, CA) in a cell incubator, which was set at 37°C and 5% CO_2_. Different levels of oxalic acid (0, 0.01, 0.05, 0.1, 0.5, and 1 mM) and/or SCFAs (0, 0.1, 1, 5, 10, and 20 mM) were added into the Caco-2 cell culture system for 24 h, and then the SLC26A3/6 expression was measured.

### Examination of renal crystals.

Three- to 4-μm sections of the kidney were prepared and stained with HE and VK according to the protocols of staining kits (Solarbio, Beijing, China). The HE staining scoring system reported by Xiang et al. (54) and the Von Kossa staining scoring method using Image Pro Plus6 software were separately applied as semiquantitative and quantitative methods to evaluate the renal crystals. Higher scores represented more crystals in the kidney.

### Analyses of urinary oxalate.

One hundred microliters of 24-h urine and 200 μl of 1,2-diaminobenzene were mixed to produce 2,3-dihydroxyquinoxaline. After centrifugation, the supernatant was used for liquid chromatography-mass spectrometry analysis using an LCMS-8040 (Shimadzu, Kyoto, Japan). The liquid chromatographic column was Shim-pack GIST-C_18_ (Shimadzu, Kyoto, Japan).

### qRT-PCR assay.

We extracted total RNAs from intestinal tissues and Caco-2 cells using an RNeasy minikit (Qiagen, Dusseldorf, Germany). Then, reverse transcription was conducted with a RevertAid first-strand cDNA synthesis kit (Thermo Scientific, Waltham, MA, USA). We performed qRT-PCR using the CFX Connect system (Bio-Rad, Hercules, CA, USA) with SYBR green (Qiagen, Dusseldorf, Germany) to examine the expression of SLC26A3/6 at mRNA level. Glyceraldehyde-3-phosphate dehydrogenase (GAPDH) was selected as the reference gene to normalize the mRNA expression. The primer sequences of GAPDH, SLC26A3, and SLC26A6 are shown in Table S1 in the supplemental material.

10.1128/mSystems.01045-21.5 TABLE S1 Primer sequences for a quantitative real-time PCR (GAPDH, glyceraldehyde-3-phosphate dehydrogenase). Download Table S1, DOCX file, 0.02 MB. Copyright © 2021 Liu et al. 2021 Liu et al. https://creativecommons.org/licenses/by/4.0/ This content is distributed under the terms of the Creative Commons Attribution 4.0 International license.

### Western blot assay.

The total protein of intestinal tissues was isolated by RIPA buffer (Beyotime, Shanghai, China) with 1% protease inhibitors and 1% phosphatase inhibitors (Thermo Scientific, Waltham, MA, USA) and boiled for 10 min at 95°C with 1× loading buffer (Biosharp, Hefei, Anhui, China). After Western electrophoresis and transfer of protein, the polyvinylidene difluoride (PVDF) membranes were blocked using Tris buffer plus Tween (TBST) containing 5% milk and incubated with β-actin primary antibody (1:5,000, ab8226; Abcam, Cambridge, United Kingdom; 1:5,000, A01011; Abbkine, Shanghai, China), SLC26A3 primary antibody (1:1,000, LS-C387170; LSBio, Seattle, WA, USA), or SLC26A6 primary antibody (1:2,500, AP32055PU-N; OriGene, Rockville, MD, USA; 1:2,500, PAB27646; Abnova, Taiwan, China). Then, the PVDF membranes were washed with TBST and incubated with anti-goat IgG (1:5,000, ab4671; Abcam, Cambridge, United Kingdom), anti‐mouse IgG (1:5,000, ab6728; Abcam, Cambridge, United Kingdom), or anti-rabbit IgG (1:5,000, ab6721; Abcam, Cambridge, United Kingdom). After washing three times, we dripped enhanced chemiluminescence reagent (Millipore, Darmstadt, Germany) on the blots and used the ChemiDoc MP imaging system (Bio-Rad, Hercules, CA, USA) for exposure. Band intensities were quantified with ImageJ (version 1.52a). Qualitative analysis was performed, and values were expressed in relation to β-actin. The relative protein expression in the control group was set as 1.

### Immunofluorescence assay.

Intestinal microsections were washed with phosphate-buffered saline with Tween (PBST) and blocked with bull serum albumin. Then, they were incubated with SLC26A3 primary antibody (1:50, sc-376187; Santa Cruz Biotechnology, Dallas, TX, USA) or SLC26A6 primary antibody (1:50, sc-515230; Santa Cruz Biotechnology, Dallas, TX, USA) at 4°C overnight. At last, they were washed with PBST and incubated with anti-rabbit antibody labeled with Alexa Fluor 594 (1:1,000, A21203; Invitrogen, Waltham, MA, USA) and 4′,6-diamino-2-phenylindole (DAPI, 1:1,000, D9542-5MG; Sigma, Darmstadt, Germany) for nuclear staining. Fluorescence intensities were quantified with ImageJ (version 1.52a).

### Measurement of cecal SCFAs.

The levels of acetate, propionate, and butyrate in cecal contents were measured using gas chromatography-mass spectrometry (GC-MS). First, 30-mg cecal samples were suspended in 90 μl of 0.5% phosphoric acid and centrifuged. The supernatant was mixed with ethyl acetate and centrifuged. Then, 4-methylvaleric acid was added into the supernatant. The mixture was analyzed with a GC-MS detector (8890B-5977B; Agilent, Santa Clara, CA, USA) and an HP-FFAP capillary column (19091F-433; Agilent, Santa Clara, CA, USA).

### Gut microbiota analyses.

The gut microbiota was analyzed through 16S rRNA gene sequencing. Briefly, the total DNA of gut microbiota was extracted from fecal samples with the QIAamp Fast DNA stool extraction kit (Qiagen, Dusseldorf, Germany). The forward primer (5′-ACTCCTACGGGAGGCAGCAG-3′) and reverse primer (5′-GGACTACHVGGGTWTCTAAT-3′) for V3-V4 regions were selected. The PCR products were extracted and purified, followed by quantifying. Then, products were sequenced using the Illumina MiSeq platform (Illumina, San Diego, CA, USA). Raw fastq files were demultiplexed and quality filtered by Trimmomatic and merged by FLASH. The threshold of similarity was set as 97% for classifying sequences into different operational taxonomic units (OTUs). The Silva (SSU132) 16S rRNA gene database was used to analyze the taxonomy of each sequence. The information on OTUs in the gut microbiota of rats was shown in Data Set S1.

10.1128/mSystems.01045-21.6 DATA SET S1 The information on OTUs in the gut microbiota of rats of control, ethylene glycol (EG), EG + Acetate, EG + Propionate, and EG + Butyrate groups and antibiotics (Abx), Abx + EG, Abx + EG + Acetate, Abx + EG + Propionate, and Abx + EG + Butyrate groups. Download Data Set S1, XLSX file, 0.3 MB. Copyright © 2021 Liu et al. 2021 Liu et al. https://creativecommons.org/licenses/by/4.0/ This content is distributed under the terms of the Creative Commons Attribution 4.0 International license.

Alpha diversity was evaluated by sobs index using Mothur (version 1.30.2). Comparative analyses of the alpha diversity index were performed using Student’s *t* test. Interindividual variability (beta diversity) at OTU level among groups was also evaluated by the PCoA, ADONIS, and ANOSIM tests. The LEfSe method was used to determine statistically different bacteria among groups. The KEGG metabolic pathways were predicted by Tax4Fun. The analysis of gut microbiota was performed with a data analysis platform (http://www.majorbio.com/).

### Statistical analysis.

Data were shown as mean ± standard deviation and analyzed with Student’s *t* test. A *P* value lower than 0.05 indicated statistical difference. The different *P* values in graphs were shown as *P* < 0.05 (*), *P* < 0.01 (**), or *P* < 0.001 (***). GraphPad Prism (version 8) was used for statistical analyses.

### Data availability.

The data are provided in the supplemental material.
