# Supplementary figures and images for: Short-Chain Fatty Acids Reduced Renal Calcium Oxalate Stones by Regulating the Expression of Intestinal Oxalate Transporter SLC26A6
Source: mSystems. 2021 Nov 16;6(6):e01045-21. doi: 10.1128/mSystems.01045-21 (PMC8594443; doi:10.1128/mSystems.01045-21)

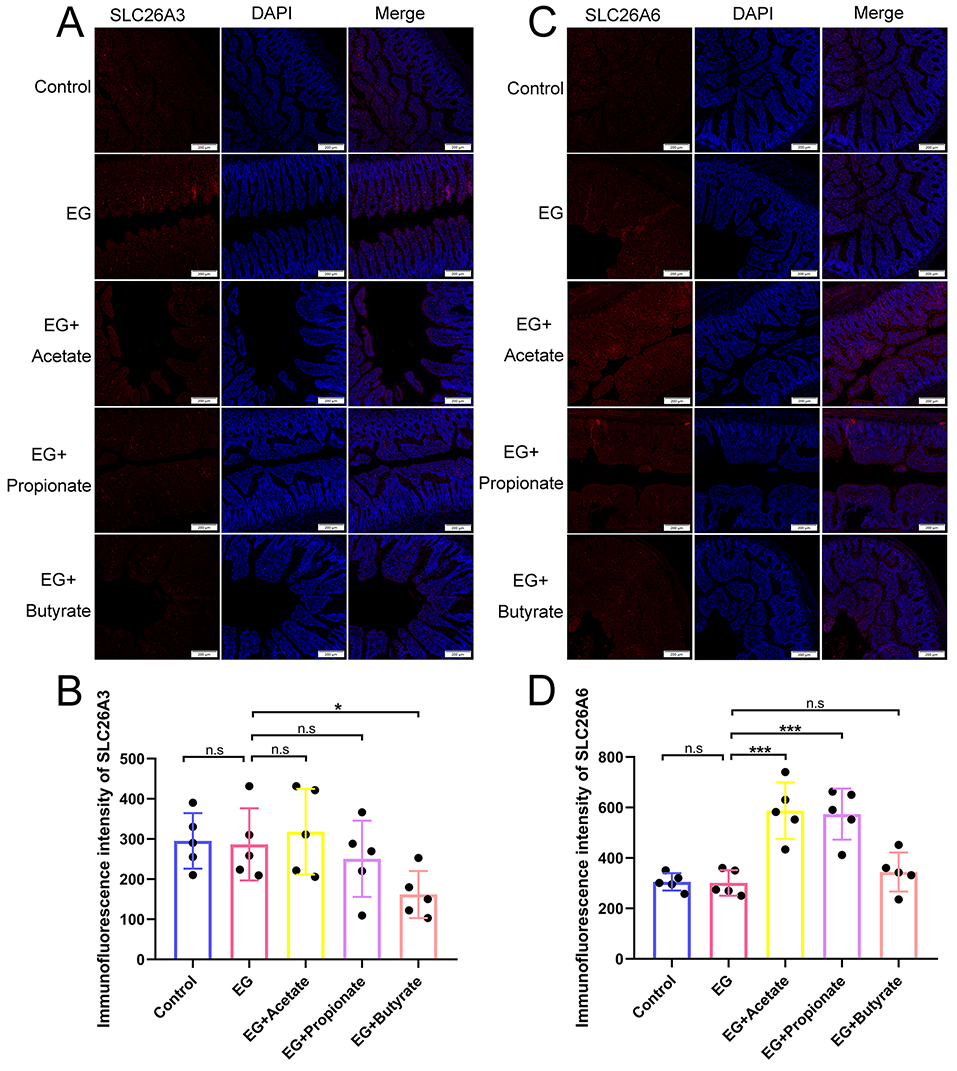

Supplement: FIG S1 [file msystems.01045-21-sf001.tif]

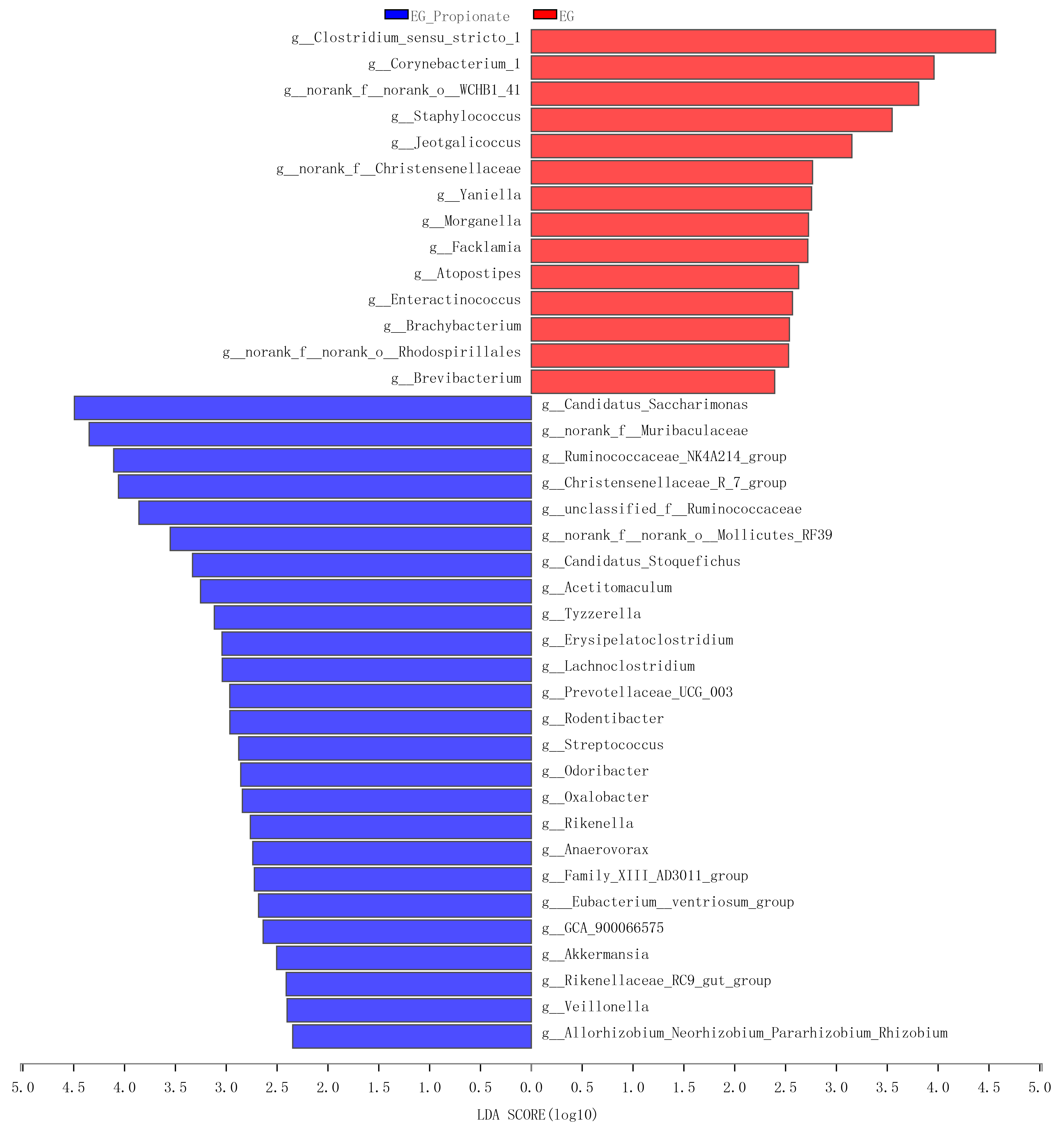

Supplement: FIG S2 [file msystems.01045-21-sf002.tif]

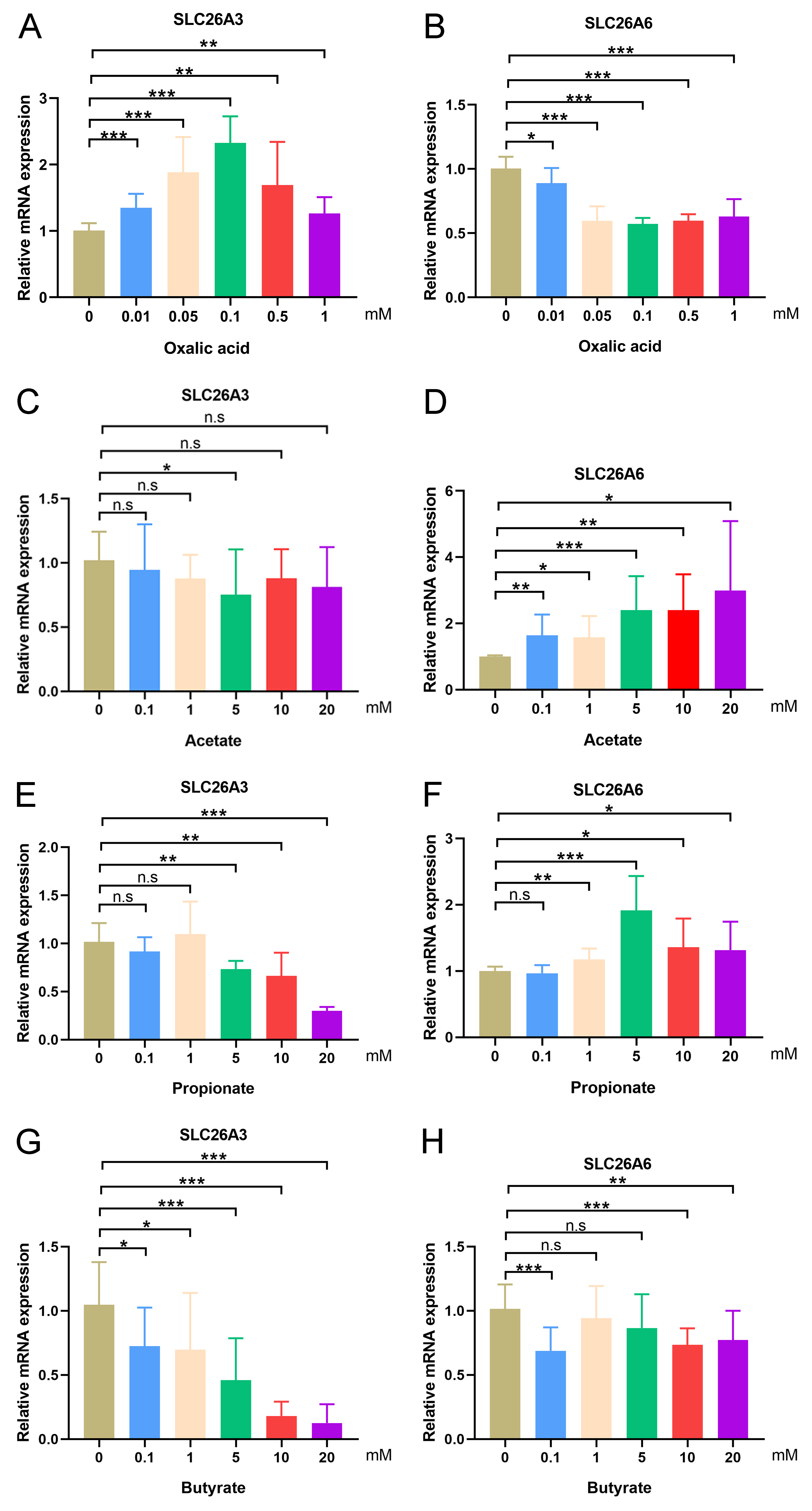

Supplement: FIG S3 [file msystems.01045-21-sf003.tif]

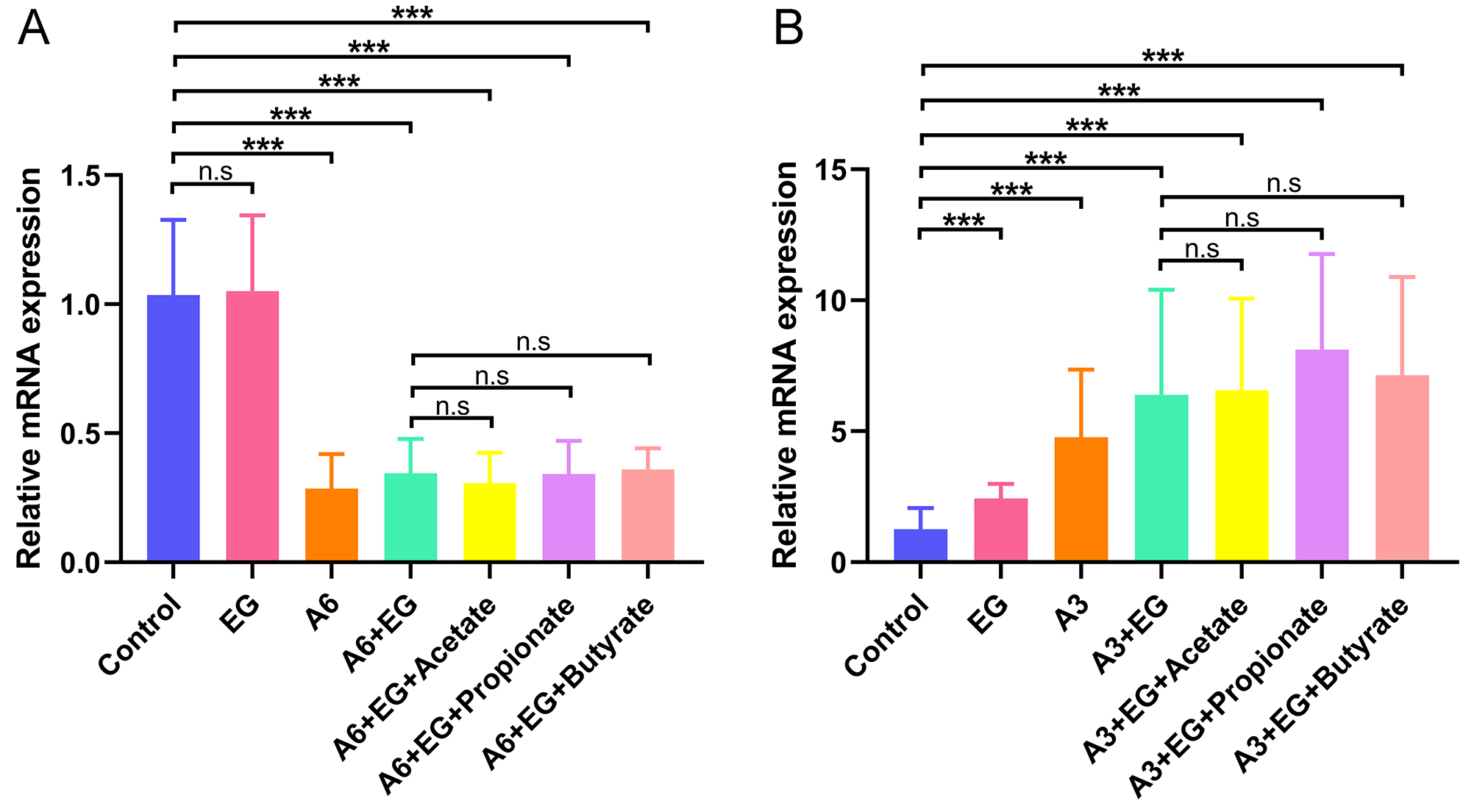

Supplement: FIG S4 [file msystems.01045-21-sf004.tif]
