# Supplementary material for: Short-Chain Fatty Acids Reduced Renal Calcium Oxalate Stones by Regulating the Expression of Intestinal Oxalate Transporter SLC26A6
Source: mSystems. 2021 Nov 16;6(6):e01045-21. doi: 10.1128/mSystems.01045-21 (PMC8594443; doi:10.1128/mSystems.01045-21)
Supplement: TABLE S1 [file msystems.01045-21-st001.docx]

**Table S1.** Primer sequences for a quantitative real-time polymerase chain reaction.

| **Species** | **Target** | **Primers (5′-3′)** |
| --- | --- | --- |
| Rat | GAPDH | F: AAGGTCGGTGTGAACGGATTTG |
|  |  | R: TGTAGTTGAGGTCAATGAAGGGGTC |
|  | SLC26A3 | F: AAAGTCTGTCCTGGCAGCTC |
|  |  | R: TGAAATGCCACACTTGCTGC |
|  | SLC26A6 | F: CAAGCCCCCAATCATGACCT |
|  |  | R: ACAGGACTGTAACAGGCTGC |
| Human | GAPDH | F: TGCACCACCAACTGCTTAGC |
|  |  | R: GGCATGGACTGTGGTCATGAG |
|  | SLC26A3 | F: TGCCAGCATACCGGCTTAAAG |
|  |  | R: TCTGGAAGTGCCGAAGAAAAG |
|  | SLC26A6 | F: CGGAGGCGAGACTACCACA |
|  |  | R: ACCAAAACCGGGAGGTGTTG |

GAPDH, glyceraldehyde-3-phosphate dehydrogenase.
